# Pathogenic Leptospira contamination in the environment: a systematic review

**DOI:** 10.1080/20008686.2024.2324820

**Published:** 2024-03-19

**Authors:** Yulia Sayanthi, Dewi Susanna

**Affiliations:** a Postgraduate Program in Public Health, Faculty of Public Health, Universitas Indonesia, Depok, Indonesia; b Department of Training-Services, PT. Immarez Solusi Utama Consultant - Training – Services, Serang, Banten, Indonesia; c Department of Environmental Health, Faculty of Public Health, Universitas Indonesia, Depok, Indonesia

**Keywords:** Leptospira, leptospirosis, environmental, soil, water

## Abstract

**Background:**

The pathogenic Leptospira is maintained in renal tubules of certain animals, mostly rodents, and excreted in the urine which can contaminate the environment. It is necessary to detect pathogenic Leptospira in environmental samples. Knowing the survival of Leptospira in the environment (water and soil) can provide an overview of where and how they can be transmitted to humans.

**Objective:**

Therefore, this study aimed to provide a systematic overview of pathogenic Leptospira presence in water and soil environment, the various species of pathogenic Leptospira that are harmful for human, and the ability to survive using a systematic review method.

**Methods:**

The search process used four databases: PubMed, Science Direct, Scopus, and ProQuest. Furthermore, the articles sought were published from 2000 to July 2021, and 38 were analysed.

**Results:**

The pathogenic Leptospira contamination in water was higher in urban areas, while soil samples were higher in rural areas. Various pathogenic Leptospira detected in the environment were L. alstonii, L. kmetyi, L. noguchii, and L. interrogans. Those pathogenic Leptospira can survive in water at 4–30°C and at pH < 7; in soil, it can survive at a humidity of < 20% and a pH < 6.

**Conclusion:**

Urban and rural areas have the same risk for leptospirosis disease because pathogenic Leptospira (P1).

## Introduction

Leptospirosis is an infectious disease caused by Leptospira that is transmitted directly or indirectly from animal to human, known as zoonosis [1]. The pathogenic Leptospira infect renal tubules of reservoir animals, mostly rodents, and shed through urine into the environment. Pathogenic Leptospira can survive in moist soil and surface water for several months [2]. Humans and other animals can be infected through their skin and mucous membranes when they encounter a contaminated environment by pathogenic Leptospira [3].

Leptospirosis outbreak is widely associated with water activities, including flooding, water sports, and work involving contact with water, such as agriculture [4]. There are 1.03 million cases worldwide, with a mortality rate of 58,900 deaths every year. The highest incidence is from Oceania (150.68 cases per 100,000 population), followed by Southeast Asia (55.54), the Caribbean (50.68), and Africa (22.65) [5]. These outbreaks have occurred in several countries such as Guyana, India, Kenya, Lao People’s Democratic Republic, New Caledonia, Nicaragua, Philippines, and Thailand and were reported to have a strong relationship with extreme weather [6]. Furthermore, 3–102 cases in Africa per 100,000 population yearly and 2.3% − 19.8% of hospitalized patients with febrile symptoms were Leptospirosis [7]. Meanwhile, in Fiji, there were 576 cases and 40 deaths from Leptospirosis after a storm that caused flooding in 2012 [8].

Leptospirosis cases in Indonesia increase every year; where in 2017, there were 894 cases, and increase in 2019 of 920 cases, with a death rate of 122 (13.26%); many cases were not reported due to its difficulty to diagnose and the high cost of Leptospirosis laboratory test [9]. The presence of rats, stagnant water around the house, and poor sewage and waste disposal conditions were significantly associated with the incidence of Leptospirosis in Indonesia [10]. A study in Gujarat reported that there was a significant relationship between the incidence of Leptospirosis and working in flooded fields (OR = 4.6, 95% CI = 1.6–17.9), as well as bathing/swimming in canals (OR = 3, 95% CI = 1.8–4.8) [11]. The risk of humans being infected with Leptospira after contact with the environment depends on the ability to survive and infect new hosts [12].

Previously, Leptospira were grouped into two pathogenic and saprophytic. Recently study found that there were 64 genera of Leptospira species and classified into four subclades, namely subclade P1 (pathogen), subclade P2 (intermediate), subclade S1 and S2 (saprophytic) [13]. The name for pathogenic Leptospira now become P1; it is the causative agent of Leptospirosis, which is distributed worldwide [14]. Pathogenic Leptospira or P1 multiply in the kidney tubules of infected mammals (hosts) [15] and excreted through urine into the environment [1]. One of the most threatening and dominant species found in human and animal is *L. interrogans*. Intermediate Leptospira have recently been discovered in humans and animals, but in animal experiments, they can not reproduce the disease. Meanwhile, the saprophyte is an environmental species non-pathogenic for humans and other animals [16]. The species belonging to subclade P1 (pathogen) are *L. interrogans*, *L. kirschneri, L. noguchii*, *L. santarosai*, *L. mayottensis*, *L. borgpetersenii*, *L. alexanderi*, and *L. weilii* [13].

Many studies have investigated the presence of Leptospira in the environmental samples. In Malaysia, from 40 samples consisting of 20 water and 20 soil samples, 5% Leptospira DNA was found in each after being tested by the qPCR (quantitative-Polymerase Chain Reaction) method [17]. A study in Jakarta took water samples from 20 flood-prone locations, and the result showed that 75% contained Leptospira saprophytes [18]. The saprophytic types live naturally on soil and water surfaces, but these species do not cause disease [3]. Meanwhile, pathogenic types live in the renal tubules of reservoir animals such as rats and then exit into the environment through urine [3].

Knowing the Leptospira’s survival in water and soil provides an overview of where and how it can be transmitted to humans [19]. However, isolation of pathogenic Leptospira (P1) was difficult because saprophytic Leptospira grows rapidly during culture, making it difficult to detect the pathogenic type. This study aimed to provide a systematic overview of pathogenic Leptospira (p1) presence in water and soil environments, the various species of pathogenic Leptospira that are harmful to humans and the ability to survive using a systematic review method.

## Methods

### Search strategy

This Systematic Review used the PRISMA stage or protocol and has been registered on PROSPERO (Id: CRD42021267260). To determine the research questions, the PICO method was used.

Population (research subject) : Leptospira in environment

Intervention : Detection of Leptospira in environmental samples by Laboratory Test (PCR)

Comparison : Rural and urban research areas (urban & rural)

Outcome : Leptospirosis

The articles were retrieved from four scientific databases, namely PubMed, Science Direct, Scopus, and ProQuest, and some were taken from the Indonesian National Journal, which is not indexed internationally. The search was conducted using the words Leptospira, environmental, water, and soil. The articles in the search database were recorded or cited using the Mendeley Program to facilitate the screening process.

The PubMed search used the keywords ‘Leptospira, environmental, water and soil’, filters applied: Full text, Journal article, English, Indonesian, from 2000/1/1–2021/7/17. ProQuest used ‘leptospiral, environmental, water, and soil’ filters applied: Academic Journal, 1 January 2000 – 17 July 2021, Articles, English. Science Direct ‘leptospira, environmental, water and soil’, 2000–2021, Refined by Article type: Research articles, Subject area: Environmental science, Medicine and Dentistry, Biochemistry, genetics and molecular. Scopus (TITLE-ABS-KEY (leptospira) AND TITLE-ABS-KEY (environmental) AND TITLE-ABS-KEY (water) AND TITLE-ABS-KEY (soil) AND (EXCLUDE (PUBYEAR, 1995) OR (EXCLUDE (PUBYEAR), 1992) OR EXCLUDE (PUBYEAR, 1991), EXCLUDE (PUBYEAR, 1973)) AND (EXCLUDE (SUBJAREA, ‘ENGI’)) AND (LIMIT-TO (DOCTYPE, ‘ar’)) AND (LIMIT-TO (LANGUAGE, ‘English’)) AND (LIMIT-TO (SRCTYPE, ‘j’).

### Inclusion and exclusion criteria

The inclusion criteria were articles published in 2000–2021 in English or Indonesian, academic articles or studies with observational designs, and environmental or experimental studies reporting the detection results of pathogenic Leptospira from the environment and/or humans. Furthermore, the criteria include articles on an environment at risk of transmitting the disease to humans and those reporting the bacteria’s survival. Meanwhile, the exclusion criteria were articles that only contained abstracts, systematic reviews, those that reported detection of pathogenic Leptospira in animals, reported detection only in humans without environmental variables, the discovery of pathogenic Leptospira in environments that are rarely touched by humans such as forests and detection of non-pathogenic Leptospira.

### Article quality assessment

The quality assessment of the articles reviewed used a list of questions that were assessed according to the information in the selected articles.

### Data extraction

The data used in the articles selected based on the inclusion criteria were the main author, year of publication, research period, the country where it was carried out, the design and methods, sampling locations and sample sources. The results related to the presence of pathogenic Leptospira in the environment were the number of samples identified, positive samples, and the species detected. Furthermore, data regarding the ability of these bacteria to survive were also analyzed.

### Data synthesis

The data were narratively synthesized, and the study area was categorized into rural, covering places outside the city with low population density, having a lot of lands such as agriculture, rice fields, plantations, livestock, as well as fields, and urban areas located in the city center with high population density.

### Overcoming bias

Two reviewers carried out the selection process according their respective fields of expertise; this is to overcome the bias in this review. When there was a conflict between the two reviewers, a third person was used as an intermediary or as a suggestion. Therefore, to overcome the bias of article synthesis, a registered review protocol with Prospero was used to improve review quality, encourage transparency, and avoid duplication.

## Results

**Figure 1.**
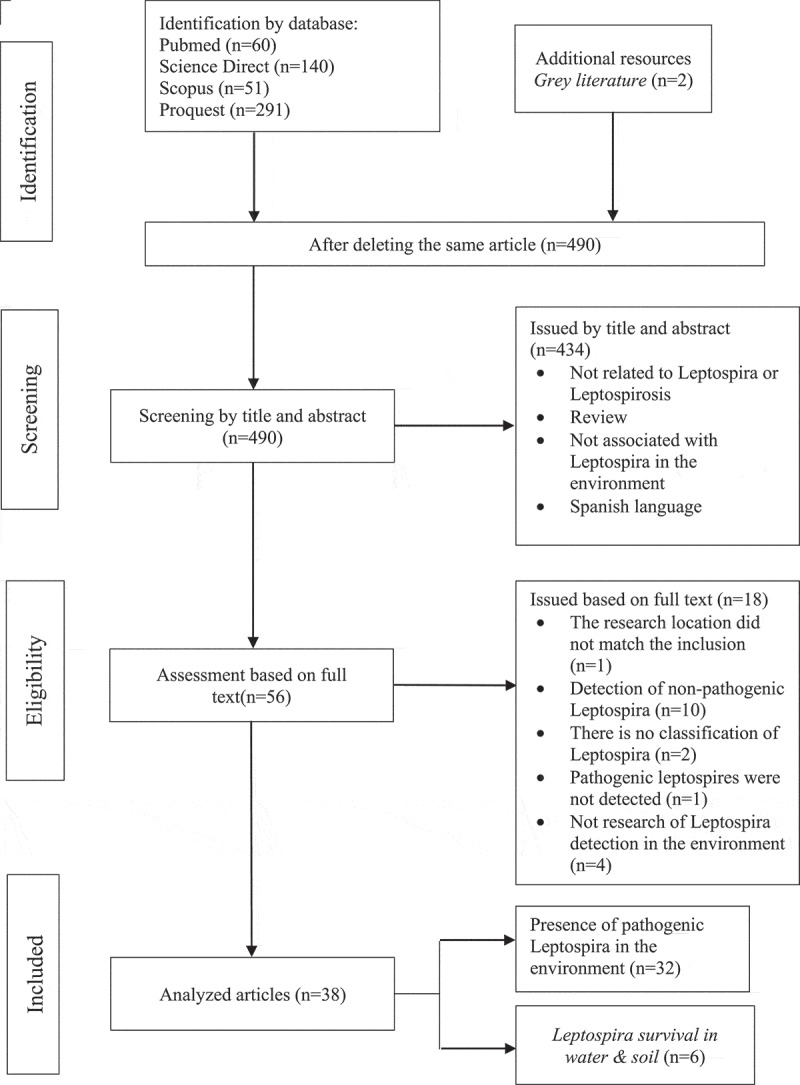
Flowchart of the article selection process based on PRISMA.

There was no risk of bias in this systematic review. Based on the article search results using four scientific databases, 542 articles were obtained, and two were added from grey literature sources. After deleting the same article, 490 were found, screened by title and abstract. Also, 434 articles were removed after being screened by title and abstract because they unrelated to the bacteria or the disease reviewed, unrelated to Leptospira in the environment, and were in Spanish. Furthermore, the full texts were reviewed to find those that matched the inclusion criteria. From the 56 articles reviewed, 18 were excluded based on the exclusion criteria, namely, the research location did not match the inclusion (*n* = 1) and detection of non-pathogenic Leptospira (*n* = 10), no Leptospira classification (*n* = 2), no pathogenic Leptospira detected (*n* = 1), and no detection study in the environment (*n* = 4). Eventually, 38 that met the inclusion criteria were analyzed. A total of 32 articles involved studies on the presence of pathogenic Leptospira in the environment, while six were about the ability to survive in water and soil media (Figure 1). Quality assessment of 38 selected articles used the scoring table, and they have moderate to good quality with an average score of 75; this information is available in the supplementary appendix.

### Article characteristics

Most of the studies came from Southeast Asia (*n* = 15), followed by South America (*n* = 8), Western Europe (*n* = 4), South Asia (*n* = 3), the United States, and Central America with two articles respectively, while West Africa, West Indies, East Asia, and Southern Europe with one article. Based on the year of publication, the most published articles were in 2018 (*n* = 6), then 2017 (*n* = 5), 2014, 2019, and 2020 with four, 2016 (*n* = 3), 2011, 2013, 2015, as well as 2021 with two articles each, and 2006, 2008, 2010, 2012 with one each. Most articles did not clearly state the type of design, but based on the method used and the time, it can be observed that the design was cross-sectional (*n* = 27), prospective (*n* = 2), case study (*n* = 2), retrospective (*n* = 1), and six articles used experimental study (Table 1).

**Table 1.** Characteristics of articles.

Variable	Number of Articles			Total
	urban	rural	urban & rural	
**Geographical Region of research origin**				
West Africa	1	-	-	1
South America	3	3	2	8
United States of America	-	2		2
Central America	2	-	-	2
West Indies	-	1		1
South Asia	-	1	2	3
Southeast Asia	5	8	2	15
East Asia		1	-	1
Western Europe	2	2	-	4
Southern Europe	1	-	-	1
Total	14	18	6	38
**Publication Year**				
2006	-	-	1	1
2008	1	-	-	1
2010	-	1	-	1
2011	-	2	-	2
2012	-	-	1	1
2013	1	1	-	2
2014	1	3	-	4
2015	1	1	-	2
2016	-	-	3	3
2017	3	2	-	5
2018	2	3	1	6
2019	2	2	-	4
2020	2	2	-	4
2021	1	1	-	2
Total	14	18	6	38
**Research design**				
*Case Study*	2	-	-	2
*Cross-Sectional*	9	14	4	27
*Experimental*	3	2	1	6
*Prospective Study*	-	1	1	2
*Retrospective Study*	-	1	-	1
Total	14	18	6	38

### Presence of Pathogenic Leptospira (P1) in water samples

The presence of pathogenic Leptospira (P1) in water samples was investigated in 30 out of 38 articles. This review compared the presence of pathogenic Leptospira (P1) in water samples based on two research areas, urban and rural. Twelve articles investigate contamination in water samples only from urban area [20–31], 14 articles only from rural areas [19,32–44], and the remaining four articles investigate in both areas [45–48]. In total, water samples from rural and urban were 2.341 samples. The percentage of contamination pathogenic Leptospira (P1) from water samples in urban was higher (18.7%) than in rural area (14.7%). The highest contamination from the urban areas was from puddles around the market in South Amerika (68%) [46]. In rural areas, the highest contamination was from water dams that are used for household activities in the West Indies (62.5%) [34].

In urban area, other Leptospira (P1) contamination from water samples were detected in public toilet 66,7% [23], water canal from slaughterhouse 16,7% [29], rice field 8,3% [26], recreational lake 1,7% [27], open drainage around household between 1,9% − 36% [20,26,28,47,48], human drinking water 8% [45], household water storage between 4% − 17,2% [21,24,25], groundwater and well between 1,6% − 33,3% [21,22,25,46]. Pathogenic Leptospira (P1) were not detected in industrial drainage [48], the puddle at slaughterhouses [29], irrigation and dry canals near the market [30].

In rural area, pathogenic Leptospira (P1) detected in spring that used for drinking 40% [34], waterfall 35,3% [36], river between 2,1%-33,3% [19,35,37,38,44,45], well 25,4% [46], household puddles between 2,2% − 41,1% [37,39,45], household water storage 17%-46,7% [42,45], drinking water 9,3%-15% [37,45], drainage from household 2.1% − 6.7% [35,48] and cowshed drainage 13,3% [48]. There was no contamination detected in the pool, stream, forest land, and river from rural area [34,48]. (Table 2)

**Table 2.** Presence of leptospira in water samples.

Geographical Region	Main Author	Sample Source	Urban					Rural					References
			Number of Samples	Positive Leptospira Pathogens			%	Number of Samples	Positive Leptospira Pathogens		%		
South America	Arnau Casanovas-Massana, 2017	Sewage Water	335	121			36.0	-	-		-		[49]
		Standing Water	250	115			46.0	-	-		-		
	Miller et al., 2021	River	-	-			-	112	4		3.6		[19]
	Mason et al., 2016	Source of animal drinking water	2	0			0	83	14		17.0		[45]
		Puddles (pool)	120	31			25.8	128	53		41.4		
		Source of human drinking water	38	3			8.0	57	11		19.3		
		Water flow	27	2			7.4	107	11		10.3		
		Water in the reservoir	53	4			7.5	141	24		17.0		
	Muñoz-zanzi et al., 2014	Puddles (ponds/rainwater containers)	-	-			-	186	36		19.3		[37]
		Canal/river	-	-			-	103	4		3.9		
		Containers (water in buckets, cans, old tires)	-	-			-	141	16		11.3		
		Animal drinking water	-	-			-	83	12		14.5		
		Human drinking water sources (wells, springs, tap water)	-	-			-	57	9		15.8		
	Ganoza et al., 2006	Puddles, sewers in the market	78	53			68.0	-	-		-		[46]
		Puddles, sewers, rainwater reservoirs, rivers, well in housing	114	38			33.3	-	-		-		
		Freshwater wells, fish farming, slow river flow	-	-			-	236	60		25.4		
	Calderón, Alfoso et al., 2013	Animal drinking water	-	-			-	18	1		5,6%		[32]
		Well	-	-			-	18	0		0%		
		Livestock wastewater	-	-			-	18	1		5,6%		
USA	Viau et al., 2011	River on the coast	-	-			-	42	4		9.5		[38]
	Verma et al., 2019	Open water (moats, ponds, puddles, rivers, springs)	-	-			-	89	2		2.2		[39]
Central America	Allwood et al., 2014	Household water storage drum	224	9			4.0	-	-		-		[24]
	Flores et al., 2020	Water storage in the house	29	1			3.4	-	-		-		[25]
		Puddle	3	0			0	-	-		-		
		Well	11	1			9.0	-	-		-		
		River	35	2			5.7	-	-		-		
South Asia	Lall et al., 2018	Puddle of rice field	-	-			-	69	8		11.6		[40]
		Field puddles	-	-			-	84	13		15.5		
		Puddles in settlements	-	-			-	32	2		6.3		
		Puddles in the forest	-	-			-	41	2		5.0		
Geographical Region	Main Author	Sample Source	Urban					Rural					References
			Number of Samples		Positive Leptospira Pathogens	%		Number of Samples	PositiveLeptospira Pathogens		%		
	Vinod et al.,2016	Rice field water	-		-	-		24	8		33.3		[47]
		Water on rice leaves	-		-	-		18	5		27.2		
		Household sewer	30		8	26.6		23	3		13.0		
		Main drain	20		7	35.0		12	2		16.6		
		Rainwater storage	6		0	0		9	1		11.1		
	Zala et al., 2018	Sewage	30		2	6.7		-	-		-		[48]
		Household drainage	20		4	20.0		-	-		-		
		Market drainage	30		1	3.3		-	-		-		
		Public toilet drainage	10		0	0		-	-		-		
		Industrial drainage	10		0	0		-	-		-		
		River	1		0	0		-	-		-		
		Rice field water	-		-	-		40	5		12.5		
		Stream	-		-	-		10	0		0		
		Household drainage	-		-	-		30	2		6.7		
		Cowshed drainage	-		-	-		30	4		13.3		
		Pool	-		-	-		1	0		0		
		Forest land	-		-	-		3	0		0		
		River	-		-	-		1	0		0		
Southeast Asia	Nugroho et al., 2017	Rice field water, puddle water, sewage water	60		5	8.3		-	-		-		[26]
	Widiastuti et al., 2015	Household water storage (washing, bathing, defecating)	-		-	-		15	7		46.7		[42]
	Azali et al., 2016	Market						36	7		19.4		[41]
		Recreation areas						36	0		0		
	Benacer et al., 2013	Lake (recreational area)	121		2	1.7		-	-		-		[27]
	Mohd Ali et al., 2018	Puddle	-		-	-		21	0		0		[43]
	Neela et al., 2019	River water around the military training ground	-		-	-		18	6		33.3		[44]
	Pui et al., 2017	Wastewater, rivers, lakes, puddles near people’s houses	324		6	1.9		-	-		-		[28]
	Ridzlan et al., 2010	Ponds, streams, drainage, puddles	-		-	-		145	3		2.1		[35]
	Zaki et al., 2020	Water flows and stagnates	-		-	-		198	26		13.3		[33]
	Tabo et al., 2019	The puddle at the slaughterhouse	6		0	0		-	-		-		[29]
		Channel	6		1	16.7		-	-		-		
		River	6		0	0		-	-		-		
		Flood water	18		0	0		-	-		-		
Geographical Region	Main Author	Sample Source	Urban					Rural					References
			Number of Samples		Positive Leptospira Pathogens	%		Number of Samples		PositiveLeptospira Pathogens		%	
	Tantengco et al., 2017	Home yard, irrigation, canal near market, paddy field, dry canal, river	97		0	0		-		-		-	[30]
	Chaiwattanarungrueng-paisan et al., 2018	Waterfall	-		-	-		17		6		35.3	[36]
	Kurilung et al., 2017	Source of groundwater & rice field water	14		3	21.4		-		-		-	[22]
East Asia	Saito et al., 2012	Water from markets or roadsides, open drainage, canals, and rivers	16		10	62.5		-		-		-	[31]
West Indies	Rawlins et al., 2014	Pool	-		-	-		15		0		0	[34]
		stream	-		-	-		3		0		0	
		Puddle	-		-	-		13		1		8	
		Dam	-		-	-		8		5		62.5	
		Springs (mountains)	-		-	-		5		2		40.0	
West Africa	Houéménou, Honoré et al., 2021	Air tanah	61		1	1,6%		-		-		-	[21]
		Kolam sementara	29		5	17,2%		-		-		-	
		Kolam permanen	17		0	0%		-		-		-	
		Air kran	83		0	0%		-		-		-	
		Danau	3		0	0%		-		-		-	
Southern Europe	Luchini et al., 2008	Air toilet	3		2	66,7%		-		-		-	[23]
		Air kran	1		0	0%		-		-		-	
		Total	2.341		437	18,7%		2.576		380		14,7%	

### Presence of Pathogenic Leptospira (P1) in soil sample

The presence of Pathogenic Leptospira (P1) in soil samples was investigated in 15 out of 38 articles. Six articles investigate contamination in soil samples only from urban areas [23,25,28,30,31,50], eight articles investigate only from rural areas [16,19,33,41,43,44,51,52], the remain 1 article investigate in both area [48]. In total, soil samples from rural and urban were 989 samples. The percentage of contamination pathogenic Leptospira (P1) from water samples in rural were higher (28%) than in urban area (14%). The highest contamination from the urban areas was from soil from a pond in the lethal case of Leptospirosis area in Southern Europe (100%) [23]. While in rural, the highest contamination was from coastal areas in Southeast Asia (47.8%) [51]. In urban areas, pathogenic Leptospira (P1) not only found around slums and poor communities [25,28,30,50], but it also found in industrial areas [48] and around campus [31]. In rural areas, contamination of pathogenic Leptospira was detected in soil samples from markets [41,48], stables [48], rice fields [48], rivers [19], beach, and coastal area [19,51], even from recreational area [16,33,41]. (Table 3)

**Table 3.** Presence of pathogenic leptospira in soil samples.

Geographical Region	Main Author	Sample Source	*urban*			*rural*			References
			Number of Samples	Positive Leptospira Pathogens	%	Number of Samples	Positive Leptospira Pathogens	%	
South America	Miller et al., 2021	river land	-	-	-	64	14	22%	[19]
		beach land	-	-	-	25	10	40%	
	Schneider, et al., 2018	The land near the rat tracks, and the ditch	70	22	31.4%	-	-	-	[50]
South Asia	Zala et al., 2018	industrial area	15	1	6.7%	-	-	-	
		Market area	10	0	0%	-	-	-	
		Land near the stables	-	-	-	15	2	13.3%	
		forest area	-	-	-	10	0	0%	
		Rice fields	-	-	-	10	1	10%	
Southeast Asia	Azali et al., 2016	Market	-	-	-	36	22	61%	[41]
		Recreation areas	-	-	-	36	4	11%	
	Mohd Ali et al., 2018	The wet soil in the patient’s house	-	-	-	21	7	33.3%	
	Neela et al., 2019	The land at the military training ground	-	-	-	18	8	44.4%	
	Pui et al., 2017	Around houses, landfills, open fields, around lakes	292	34	11.6%	-	-	-	
	Zaki et al., 2020	Recreation area (topsoil)	-	-	-	198	26	13%	
East Asia	Saito et al., 2012	Around Kyusu campus	12	3	25%	-	-	-	[31]
Western Europe	Thibeaux, et al., 2017	Recreational areas (where the patient is exposed)	-	-	-	52	30	58%	[16]
Central America	Flores et al., 2020	Rain puddle land	20	1	5%	-	-	-	[25]
		River	22	2	9%	-	-	-	
Southeast Asia	Saito et al., 2014	Wetlands on the coast	-	-	-	23	11	47.8%	[51]
	Tatengco et al., 2017	Around the trash	38	3	8%	-	-	-	[30]
Southern Erope	Luchini et al., 2008	Around the pool	2	2	100%	-	-	-	[23]
East Asia	Fuh, Ying Bin, et al., 2011	Cow and chicken farm	-	-	-	108	7	6.5	[52]
		Total	481	68	14%	508	142	28%	

### Identification results of Pathogenic Leptospira (P1) species in water and soil samples

Various Leptospira species that are harmful and able to infect animals (agent) and humans were detected in water and soil samples. They are *L. alstonii, L. kmetyi, L. noguchii*, and *L. interrogans*. Those species were detected in a rural and urban settings.

### Pathogenic Leptospira (P1) survival ability in environmental samples

Pathogenic Leptospira (P1) can survive in soil with humidity < 20% [31] and can survive for four days in the soil after a storm [51] but cannot reproduce in the environment [49]. It is still able to infect even in poor nutritional conditions [6] and can survive in low pH water for at least 20 months [53] as well as in nutrient-free water for more than a year [6]. In addition, they can outlive and move faster or slower than the average speed of flowing water [54] (Table 4).

**Table 4.** Leptospira survival in the environmental samples.

Main Author	Microorganism	Environmental condition	Findings	References
Saito et al.. (2012)	All variant	Moisture content of soil samples between 3,4% − 42,8%,	Leptospira survive in the soil (67%) with moisture content of > 20%.	[31]
		pH value of soil samples between 6,2–7,2	pH value higher than 6,2 had no influence to the distribution and survival of Leptospira in the soil	
Saito et al. (2014)	Pathogenic Leptospira	Artificial seawater (sodium chloride)	Leptospira were killed within several hours	[51]
		Seawater	Leptospira survive approximately 3 days	
		Isolate mix with soil	Leptospira survive in seawater for 4 days	
			These results suggest that leptospires living in soil are more resistant to seawater	
Cassanovas-Massana et al. (2018)	*L. interrogansC*openhageni	Laboratory microcosms	The findings suggest that the environment is not a multiplication reservoir but a temporary carrier of *L. interrogans* Copenhageni, although the observed prolonged persistence at low concentrations may still enable the transmission of Leptospirosis	[49]
Andre-Fontaine (2015)	*Leptospira spp.*	Water samples that were stored for 20 months at 4°C, 20°C or 30°C. The survival and preservation of virulence of Leptospira spp. was estimated by subculturing these stored samples.	The finding of this experiment was showed Leptospira spp. that despite unfavourable storage conditions such as cold, nutrient-poor acidic waters, the survival and virulence of pathogenic Leptospira spp. was fully preserved over at least 20 months.	[53]
Nau et al. (2020)	*L. kirschneri*	50-meter-long hose system simulating a water stream	From this experiment results concluded that once excreted via animal urine, the leptospires immediately need moisture or a water body to survive and stay infectious.	[54]
Bierque et al.. (2020)	*Leptospira interrogans*	water microcosms were designed to evaluate the survival and virulence of Leptospira spp. for 2 years using four commercial bottled drinking waters and a non-ionized water, all previously filter-sterilized.	The results confirmed that pathogenic Leptospira able to survive for more than a year in water. In addition, showed the ability of *L. interrogans* in nutrient-deprived conditions to directly cause systemic infection in susceptible animals.	[55]

## Discussion

Pathogenic Leptospira (P1) live and reproduce in the kidney tubules of animal, mostly rodents, and excreted into the environment through the urine of infected animals. Survival of pathogenic Leptospira (P1) in the environment after being shed via animal urine is a key factor in estimating the risk of infection. This review showed that once pathogenic Leptospira (P1)is excreted into the environment through the urine of the infected animal, Leptospira immediately needs to be in a humid environment or body of water to survive and remain capable of infecting pathogenic Leptospira (P1) could not survive on hard surface [54].

Pathogenic Leptospira (P1) can survive in wet soil during dry days and emerge to the surface during rainy days. Furthermore, it can survive in soil with a humidity of < 20% and pH levels have no effect on distribution and survival in the soil [31]. Pathogenic Leptospira (P1) only survived 12 hours in sodium chloride, while it lasted for three days in natural seawater [51]. Meanwhile, freshwater pathogenic Leptospira (P1) survived for more than one year or at least 20 months, and even under conditions of nutritional deficiency, it still can infect susceptible animals [6].

Pathogenic Leptospira can not multiply in the environment [49]. Based on a study using water with different temperatures and pH levels, it is known that pathogenic Leptospira (P1) is able to survive at 4°C for 130 days, at 20°C for 236 days, and at 30°C for 316 days. Based on pH levels, pathogenic Leptospira (P1) survived for 344 days at pH 7 while at pH < 7 for 129 days. Pathogenic Leptospira (P1) is also capable of infecting experimental animals even though the pH of the water was reduced to < 6. Therefore, it was concluded that even at cold temperatures and low levels of nutrients in the water, pathogenic Leptospira was able to survive and become infected for at least 20 months [53].

Pathogenic Leptospira (P1) was rarely detected in soil samples because it prefers water bodies or very humid environments. In this review, the proportion of soil samples was less than that of water samples. However, the presence of pathogenic Leptospira (P1) in soil samples has been proven; it is important to be known to increase awareness and prevention of Leptospirosis transmission. To detect Leptospira in soil samples, it is necessary to pay attention to sampling location, which should include wet, mud, or humus soil. The samples were obtained in wet and shady places [16,25]. Pathogenic Leptospira (P1) contamination in soil samples from urban areas is mostly found in industrial and recreational areas, while in rural areas, they are more commonly found around livestock, rice fields, and the patient’s home [16,52].

The presence of pathogenic Leptospira (P1) in soil samples was not related to environmental characteristic such as vegetation, rat density, distance from open sewers to houses, or clay content. However, samples with high water content showed a high prevalence [50]. The survival of pathogenic Leptospira (P1) in the soil influenced by the nutrients contained, such as iron, manganese, and copper. The major content is iron, which helps to maintain the virulence and growth of pathogenic Leptospira (P1) [40]. The study in New Caledonia reported that pathogenic Leptospira (P1) was detected in soil samples from the site of suspected exposure at nine weeks from the estimated time, but not after 12 weeks [16].

People who work in agriculture, farmers, veterinarians, forest surveyors, mining, and laboratory workers are the high risk of Leptospirosis because pathogenic Leptospira (P1) is able to survive in wet soil (aquatic ecosystem) [47,56,57]. To avoid transmission of leptospirosis, the worker is recommended to wear special clothing, such as boots, masks, and gloves, to avoid contact with the skin or soil or materials that have been contaminated. After carrying out the work, laboratory workers and abattoirs are advised to wash work tools with sodium hypochlorite in a dilution of 1:4000 or use detergents [58].

Pathogenic Leptospira (P1) species detected in water and soil samples both in urban and rural areas, there were *L. alstonii* [27,31,36,41], *L. kmetyi* [30,33,36,43,44,51], *L. interrogans* [16,22,28], *L. weilii* [45,59], and *L. noguchii* [28]. *L. interrogans* and *L. kirschneri* are the most infectious and harmful for humans [59,60]. Even *L. wolffii* from the intermediate group (P2) has also been found in human samples. The P2 was dominant in environmental samples. This condition emphasizes that the environment is a high-risk factor for transmission [22]. A study in Thailand and Colombia detected *L. interrogans* in humans, pigs, dogs, and water samples. The water samples containing *L. interrogans* came from livestock drinking water sources and sewage. Many cases in rural areas of Leptospira were unreported because some infected humans did not show any symptoms [22,32]. Furthermore, studies in Italy found *L*. *interrogans* in public toilet. This investigation was carried out after a 65-year-old toilet worker was found dead, and *L. interrogans* was found in his body [23].

Leptospirosis is commonly known as a disease that originates from water; the presence of pathogenic Leptospira (P1) in the soil cannot be ignored. Therefore, knowledge of environmental risk factors, especially in the soil, can prevent transmission in risky environments such as markets, agriculture, livestock, and natural recreational areas.

### Limitations

The limitation of this study was that it could not perform a meta-analysis due to the limited quantitative data available in the articles obtained. The information was obtained from primary reports sourced from reliable databases. For further study, when the bacteria in water samples in urban areas are to be investigated, the samples should be taken from open household sewers, rainwater, or floodwaters. In rural areas, it should be obtained from wells, rain-fed water, other open reservoirs, and surface water around the house, such as rivers or rainwater puddles. When Leptospira in soil samples is being investigated, it should be carried out on the soil around the case’s house, and the time for environmental sampling should be 1–2 months after infection. In addition, the soil sample should be wet and taken in a shady location.

## Conclusion

Our review suggests that pathogenic Leptospira (P1) spreads in the environment and is able to survive in the water body for months and in moist soil for a few days. Urban and rural areas have the same risk for leptospirosis disease because pathogenic Leptospira (P1) was detected in both areas. The contamination of pathogenic Leptospira (P1) was not only detected in slums or poor household areas, but it was also detected in industrial and recreational areas. This information could be used to increase awareness and prevent the transmission of leptospirosis.

## Supplementary Material

Supplemental Material
